# Evaluation of the efficacy and safety of acupuncture assisted treatment for atrial fibrillation: A systematic review and meta-analysis based on randomized controlled trials

**DOI:** 10.1097/MD.0000000000040474

**Published:** 2024-11-29

**Authors:** Yuqing Liu, Xuemeng Pang, Yajuan Wang, Xu Liu, Hongju Jiang

**Affiliations:** a Shandong University of Traditional Chinese Medicine, First Clinical Medical College, Jinan City, Shandong Province, China; b Shandong Provincial Third Hospital, Jinan City, Shandong Province, China; c Shandong University of Traditional Chinese Medicine, Second Clinical Medical College, Jinan City, Shandong Province, China; d The Second Affiliated Hospital of Shandong University of Traditional Chinese Medicine, Jinan City, Shandong Province, China.

**Keywords:** acupuncture, atrial fibrillation, meta-analysis

## Abstract

**Background:**

To systematically evaluate the efficacy and safety of acupuncture in the treatment of atrial fibrillation (AF).

**Methods:**

Eight databases were searched. The search time limit is from January 2000 to November 2023. All randomized controlled trials on acupuncture treatment of AF were included. After the literature screening, data extraction and quality evaluation were carried out independently according to the inclusion and exclusion criteria, and the included literature was analyzed by Meta using RevMan 5.4 software.

**Results:**

A total of 15 research studies on randomized controlled trials were included, involving 1960 patients. The results of the meta-analysis showed that acupuncture therapy could increase the sinus cardioversion rate of patients with AF, and the difference was statistically significant (relative risk = 1.21, 25% confidence interval (CI) [1.11, 1.31], *P* < .001). The clinically effective rate of the acupuncture plus drug treatment group was higher than that of the drug treatment group (relative risk = 1.32, 95% CI [1.19, 1.46], *P* < .01). Acupuncture plus other conventional therapies treatment was more helpful in reducing the ventricular rate of patients with AF (mean difference = -7.89, 95% CI [-14.52, -1.26], *P* = .006). The cardioversion time of patients with AF treated with acupuncture plus conventional therapies was shorter than those treated with traditional therapies alone (standardized mean difference = -1.82, 95% CI [-3.28, -0.35], *P* = .01). No severe adverse reactions such as hemorrhage, hematoma, or local infection caused by acupuncture were reported in the study.

**Conclusion:**

The available evidence shows that acupuncture can effectively improve the total clinical effective rate and sinus rhythm recovery rate, shorten the recovery time of sinus rhythm, and reduce the ventricular rate, and there are no apparent adverse reactions. However, a limited number of studies may affect the generalizability of the findings. Future studies should include more extensive and diverse studies to enhance the power and generalizability of the findings.

## 1. Introduction

In recent years, the incidence of atrial fibrillation (AF) has a gradually increasing trend.^[1,2]^ As of 2010, there were about 33.5 million patients with AF worldwide. In China, the prevalence rate can be as high as 0.77% and gradually increases with age.^[3–5]^ AF can cause many other serious complications, among which the risk of ischemic stroke in patients with AF is 4 to 5 times higher than that in patients without AF, resulting in nearly 20% mortality and 60% disability.^[6]^ The lifetime risk of AF in patients over 40 years old was 26% in males and 23% in females.^[7]^ AF can lead to 2 times increase in all-cause mortality in women and 1.5 times in men. The leading causes of death are progressive heart failure, cardiac arrest, and stroke.^[8]^

Antiarrhythmic drugs are stable in the treatment of paroxysmal AF, but the symptom improvement rate for persistent AF is only 30%.^[9]^ At the same time, although flecainide and propafenone exhibit a lower risk for adverse events compared to amiodarone, extracardiac adverse reactions are associated. As a first-line drug, the adverse reaction of β-blocker is less than that of antiarrhythmic drugs, but the effect of maintaining sinus rhythm is weak.^[10]^ Catheter ablation (CA) is a method of rhythm control through invasive procedures. The success rate of paroxysmal AF is stable at 60% to 70%,^[11]^ while the success rate of patients with persistent AF is only 45% to 60% within 1 year after operation.^[12,13]^ Many patients need to CA again.

The main ways of modern medical treatment of AF include antithrombotic therapy, ventricular rate control, and rhythm control. Although the symptoms can be controlled quickly, the limitations are also apparent. In 2000, the terminal group of AF of the European College of Cardiology emphasized that the quality of life of patients with AF was regarded as an essential index in the evaluation outcome of AF treatment.^[13–16]^ Therefore, evaluating patients’ quality of life with AF is a hot issue for medical workers. Prevention of thromboembolism and rhythm control to restore sinus rhythm and ventricular rate are the keys to the treatment of AF.^[14]^

As the most commonly used treatment in traditional Chinese medicine, acupuncture is widely used in the prevention and treatment of arrhythmias. Studies have shown that acupuncture assisted treatment of AF can further control the ventricular rate, increase the conversion rate, and reduce the recurrence.^[10,14–17]^ Some studies also prove that acupuncture could reduce the recurrence rate of AF after CA and improve the quality of life of patients.^[15]^ Recent studies have confirmed that acupuncture, a cost-effective and environmentally friendly therapy recognized by traditional medicine, is used in the clinical treatment of AF.^[18,19]^ It is safe and effective in relieving AF symptoms and reducing recurrence rates after early radiofrequency CA and electrical cardioversion. Acupuncture can also improve sleep and ease anxiety and depression in patients with AF,^[20]^ improving their quality of life. Studies have confirmed that acupuncture therapy can treat AF by improving atrial structural remodeling, electrical remodeling, autonomic nervous system dysfunction, and abnormal calcium ion regulation.^[21]^ While specific studies have demonstrated the efficacy of acupuncture in treating AF, there has yet to be a systematic analysis to validate its effectiveness and variability in this context.

In this study, we summarize the clinical literature related to the treatment of AF with acupuncture. We included more recent studies and analyzed data from more databases in different countries. The treatment of AF was systematically evaluated, and meta analyzed some important therapeutic indexes.

## 2. Method

This study followed the Preferred Reporting Items for Systematic Reviews and Meta-Analyses (PRISMA) guidelines.^[22]^ For this meta-analysis, we used randomized controlled trials (RCTs) and studies on acupuncture for AF. This systematic review and meta-analysis was registered in PROSPERO, an international prospective register of systematic reviews, with the registration number CRD42024594746 (available from https://www.crd.york.ac.uk/prospero/). This study does not involve human experiments or clinical research and animal experiments, so it does not require the review and approval of the ethics committee, nor does it need to provide written informed consent to participate in this research.

### 2.1. Search strategy

Infrastructure (CNKI), China Science and Technology Journal (VIP) database, and Wan Fang database, were searched for RCTs based on acupuncture for AF. The search period is from the start date of each database to November 1, 2023. The retrieval strategy uses a combination of topic words and free words based on the PICOS principles (overall, intervention, comparison, outcome, and research design). Chinese search terms include AF, acupuncture and moxibustion, meridians, clinical research, clinical trials, controlled clinical trials, RCTs, and practical clinical trials. The English search terms for WOS are as follows:

#1 TH = (Atrial Fibrillation OR Atrial Fibrillations OR Fibrillation, Atrial OR Fibrillations, Atrial OR Auricular Fibrillation OR Auricular Fibrillations OR Fibrillation, Auricular OR Fibrillations, Auricular OR Persistent Atrial Fibrillation OR Atrial Fibrillation, Persistent OR Atrial Fibrillations, Persistent OR Fibrillation, Persistent Atrial OR Fibrillations, Persistent Atrial OR Persistent Atrial Fibrillations OR Familial Atrial Fibrillation OR Atrial Fibrillation, Familial OR Familial Atrial Fibrillations OR Atrial Fibrillations, Familial OR Fibrillation, Familial Atrial OR Fibrillations, Familial Atrial OR Paroxysmal Atrial Fibrillation OR Atrial Fibrillation, Paroxysmal OR Atrial Fibrillations, Paroxysmal OR Fibrillation, Paroxysmal Atrial OR Fibrillations, Paroxysmal Atrial OR Paroxysmal Atrial Fibrillations).

#2 TH = (Acupuncture OR needling OR Pharmacopuncture OR acupoint).

#3 #1 AND #2.

### 2.2. Inclusion criteria

Eight databases, including PubMed, Web of Science, Cochrane, Embase, China Biomedical Literature System, and China National Knowledge Inclusion criteria, were formulated based on the PICOS framework.

(1) Research type: RCT, sample size ≥ 10 cases. Since the evaluation treatment is acupuncture therapy, it is not easy to achieve double-blindness. According to Cochrane convention, the included studies have no restriction on whether to use blinding.
(2) Participants: patients with AF, including paroxysmal and persistent AF.
(3) Intervention measures: the intervention measures for the experimental group were acupuncture at specific acupoints and medication or CA, while the control group was treated with medication alone or sham acupuncture. There were no restrictions on the use of antiarrhythmic drugs in each research institute and no restrictions on the time and duration of acupuncture treatment.
(4) Outcome measure: the leading indicator is clinical efficacy. In the criteria for determining efficacy, efficacy refers to symptom relief, disappearance, and restoration of AF to sinus rhythm or ventricular rate control at 70–90 per minute. Ineffectiveness is characterized by no relief of symptoms, persistent AF, and a ventricular rate >100 per minute. Secondary outcome measures include sinus rhythm conversion rate, ventricular rate, and conversion time.

### 2.3. Exclusion criteria

(1) Repetitive literature.
(2) Review, expert experience summary, and theoretical exposition of non-RCT research.
(3) Animal experiments, adverse reaction reports, case reports, and other non-clinical trial studies.
(4) Retrospective or single-arm clinical studies.

### 2.4. Literature screening, data extraction

The literature mainly includes the author’s name, publication year, sample size, age, gender, acupuncture time, etc. The results extracted from the study are outcome indicators included in the literature. According to the extracted data, the acupuncture time is divided into < 20 minutes, 20 to 30 minutes, and >30 minutes. Two researchers independently extracted general information, acupuncture methods, treatment courses, outcome indicators, adverse reactions, and other data of patients in each study using a unified data extraction table. In disagreements, the 2 primary reviewers consulted with a third reviewer to achieve consensus. Statistical analysis of data was conducted using RevMan5.4 software provided by the Cochrane Collaboration Network. Qualitative data is represented by risk ratio (RR), while continuous variables are represented by mean deviation (MD) to indicate treatment effects. Both are represented by effect values and 95% confidence intervals (CI). All data were analyzed using a fixed or random effects model for meta-analysis. Perform funnel plot analysis on the included literature to evaluate whether there may be publication bias.

### 2.5. Quality assessment

The methodological quality of the included studies was evaluated using the “Risk of Bias Assessment Tool” from RCT in section 5.4 of the Cochrane System Evaluator Handbook.^[22]^ This tool comprises 7 domains: adequate sequence generation, allocation concealment, participant and personnel blinding, outcome assessment blinding, incomplete outcome data, selective reporting, and other biases. Each question is assessed according to the following criteria: “Yes” (+) indicates a low risk of bias, “Unclear” (?) suggests an ambiguous risk of bias, and “No” (-) denotes a high risk of bias. The study is considered low risk if the method used is appropriate, correct, and clearly described. Otherwise, if the process cannot accurately be determined, it will be rated as high risk, or it may be unclear. Two reviewers independently assessed the risk of bias in the included RCTs.

### 2.6. Sensitivity analysis

Sensitivity analysis is performed based on the characteristics of the studies included in the research. This involves excluding certain low-quality studies or those employing different efficacy evaluation criteria and inclusion/exclusion criteria. Subsequently, a combined analysis is conducted to compare the combined effect size before and after exclusion, thereby exploring the impact of the excluded studies on the overall effect size and enhancing the robustness of the meta-analysis.

### 2.7. Publication bias

By constructing a funnel plot, assess the symmetry of the data distribution. If the funnel plot presents a symmetrical shape, it usually indicates no significant publication bias; if asymmetric, there may be bias.

### 2.8. Statistical analysis

Use Revman (v5.4) to process literature data. The RR and its 95% CI were computed for dichotomous variables. Continuous variables were analyzed using the MD or standardized mean difference (SMD) with their associated 95% CIs. The meta-analysis follows strict PRISMA guidelines and uses *P*-values and I^2^ for heterogeneity testing. If there is no statistical heterogeneity between these research results (I^2^ ≤ 40%, *P* > .1), a fixed-effects model (using the M–H method for binary variables) is chosen. If there is statistical heterogeneity between studies, metaregression or subgroup analysis will be used to explore the sources of heterogeneity further. If the sources of heterogeneity are unclear, a random effects model (D + L method) will be used for analysis.

## 3. Results

### 3.1. Study selection

**Figure 1.**
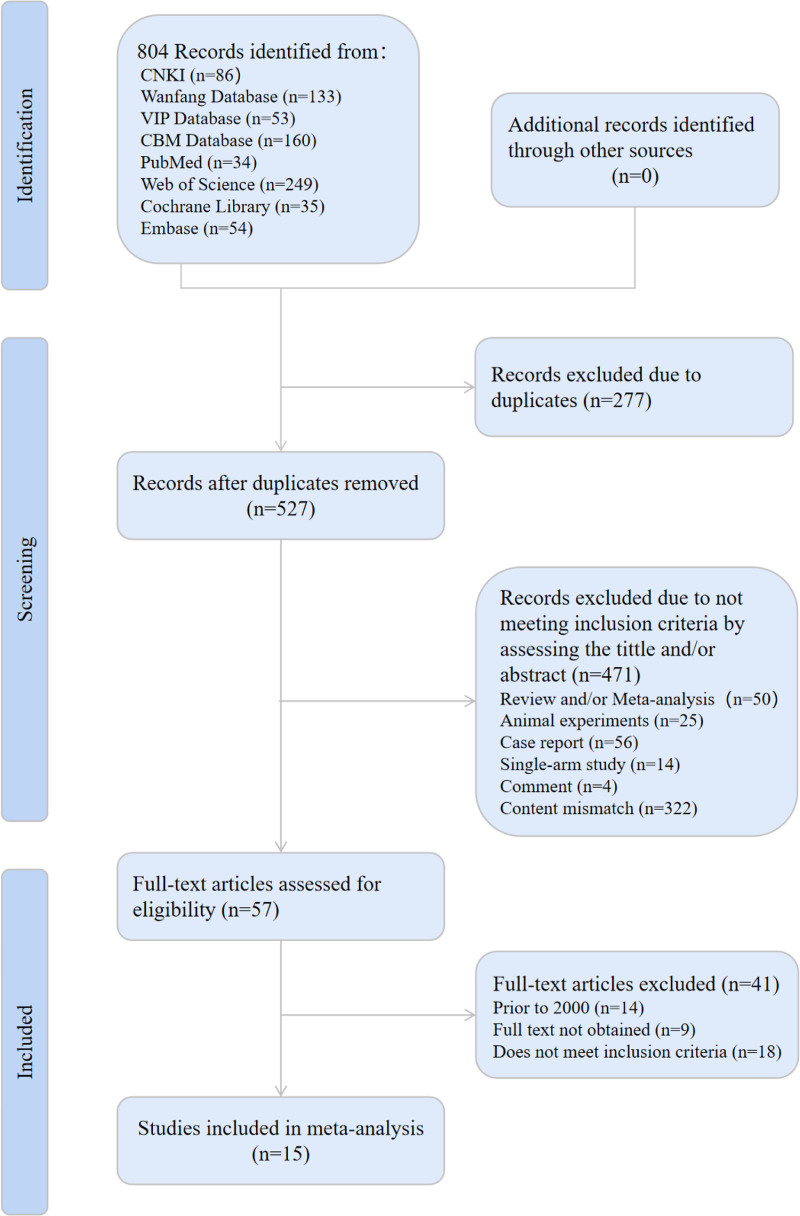
Flow diagram for selected literature.

A total of 804 articles were identified in the 8 databases, including 432 in Chinese and 372 in English: MEDLINE/PubMed (n = 34), EMBASE (n = 54), Cochrane Central Register of Controlled Trials (n = 35), CNKI (n = 86), Wanfang Database (n = 133), VIP Database (n = 53), and CBM Database (n = 160). There were 277 duplicated articles,50 reviews and meta-analyses, 4 comments, 56 case reports, 25 animal experiments, 14 non-RCT articles, and 322 non-acupuncture interventions; after reading the full text carefully, the data of 9 articles were incomplete, 18 articles did not meet inclusion criteria, and 14 articles were excluded because of out of time. Finally, 15 RCTs were included, involving 1960 patients.^[15,17,23–35]^ The literature screening process is illustrated in Figure 1.

### 3.2. Characteristics of the included studies

All these papers were published between 2000 and 2023, exclusively in English and Chinese. Based on data derived from these fifteen RCTs, the sample sizes of the studies varied, ranging from 30 to 114 participants. All participants were aged between 51.7 ± 5.8 to 70.82 ± 8.73 years. In views of types of AF: 8 studies^[24–30,33]^ included paroxysmal AF;4 studies^[17,23,31,35]^ included persistent AF and 3 studies^[15,32,34]^ included both paroxysmal and persistent AF. In terms of treatment methodology, 1 study^[34]^ exclusively employed needle pricking therapy; 1 study^[28]^ only used auricular acupoint acupuncture therapy^[23]^; 8 studies^[24–31]^ combined needle pricking therapy with drugs; 2 studies^[15,35]^ combined needle pricking therapy with CA; 2 study^[17,32]^ integrated needle therapy with CA and drugs, 1 studies^[23]^ employed electroacupuncture and electrical cardioversion. Generally, the duration of tuina treatment in studies ranges from 15 to 60 minutes, and auricular acupoint acupuncture for 24-hour treatment. The details of the study characteristics are presented in Table 1.

**Table 1:** Characteristics of the included clinical studies.

Study	Participants		Gender (male %)		Age (mean ± SD)		BMI/BSA (kg/m^2^)		CHA2DS2-VASC score		LVEF (%)		LAD (mm)	
	Trail	Control	Trail	Control	Trail	Control	Trail	Control	Trail	Control	Trail	Control	Trail	Control
Junkui Yin (2019)	40	45	62.5	62.2	61.6 ± 10.6	62.6 ± 9.1	24.0 ± 2.1	24.5 ± 2.4	1.9 ± 1.5	2.0 ± 1.1	55.0 ± 2.9	55.1 ± 3.3	44.1 ± 5.8	43.2 ± 4.5
Baozhen Xu (2015)	54	54	55.6	51.9	63 ± 7	64 ± 7	–	–	–	–	–	–	–	–
Baode Han (2012)	62	52	56.5	55.8	64.3 (48–77)	65.7 (47–75)	–	–	–	–	–	–	–	–
Yahong Yan (2014)	30	30	60	53.3	52.±6.7	51.7 ± 5.8	–	–	–	–	–	–	–	–
Li Chen (2012)	30	30	53.3	53.3	62.3 ± 5.4	64.1 ± 6.2	–	–	–	–	–	–	–	–
Hongke Xu (2007)	40	40	62.4	67.5	58.9 ± 10.5	57.4 ± 9.4	–	–	–	–	–	–	38.1 ± 5.1	37.9 ± 6.5
Xuelian Zhang (2013)	30	30	63.3	53.3	59 ± 4	55 ± 7	–	–	–	–	–	–	–	–
Jieqiong Zhang (2020)	34	34	61.8	58.8	61.2 ± 5.4	60.8 ± 5.1	–	–	–	–	–	–	–	–
Jianbao Wang (2022)	47	47	63.8	59.6	61.6 ± 11.3	61.5 ± 11.3	–	–	–	–	45.4 ± 4.2	45.0 ± 4.2	–	–
Yang Chu (2023)	31	33	61.3	69.7	68.87 ± 9.36	70.82 ± 8.73	–	–	2.55 ± 1.5	3.06 ± 1.2	58.3 ± 5.8	60.0 ± 3.9	43.42 ± 3.91	42.00 ± 3.4
Hewen Li (2023)	25	27	66.7	80.0	66.0 (51.8, 70.0)	65.0 (57.0, 70.0)	25.41 ± 3.48	26.86 ± 3.06	0:6 (20.0%) 1:6 (20.0%) 2:18 (60.0%)	0:7 (23.3%) 1:6 (20.0%) 2:17 (56.7%)	61.4 (57.2, 63.8)	61.6 (57.8, 65.0)	40.0 (35.8, 43.5)	42.00 (37.5, 45.3)
Xiaofei Gao (2023)	50	50	52.0	64.0	62 ± 12	62 ± 12	24.70 ± 3.72	24.20 ± 3.13	–	–	–	–	–	–
Albert Lomuscio (2011)	17	13	58.8	76.9	65 (62–67)	63 (59–66)	–	–	–	–	58 (55–60)	56 (52–61)	40 (39–42)	40 (37–43)
Jung Myung Lee (2021)	16	15	81.3	80.0	69.6 ± 7.3	63.7 ± 6.5	1.8 ± 0.2 (BSA)	1.8 ± 0.2 (BSA)	2.8 ± 1.5	1.6 ± 1.0	61.0 ± 6.8	64.2 ± 5.3	–	–
Yuanshi Xia (2014)	50	40	53.3		62.8 ± 5.5		–	–	–	–	–	–	–	–

Study	AF duration		Classification				Intervention				Duration of treatment		Acupoint	
	Trail	Control					Trail		Control					
Junkui Yin (2019)	14.2 ± 17.0 m	21.1 ± 29.1 m	Persistent				Needle pricking + Amiodarone		Amiodarone		20 minutes		Neiguan (PC6)	
Baozhen Xu (2015)	5. 7 ± 1. 0 years	5. 6 ± 1. 1 year	Paroxysm				Needle pricking + Wenxin Granules		Wenxin Granules		30 minutes		Neiguan (PC6) Ximen (PC4) Xuehai (SP10) Sanyinjiao (SP6)	
Baode Han (2012)	–	–	Paroxysm				Needle pricking + Digilanid C		Digilanid C		60 minutes		Neiguan (PC6) Ximen (PC4) Xuehai (SP10) Sanyinjiao (SP6)	
Yahong Yan (2014)	5.65 ± 1.6 m	5.48 ± 2.1 m	Paroxysm				Needle pricking + Meglumine adenosine cyclophosphate + Shuxuening injection + Digoxin		Meglumine adenosine cyclophosphate + Shuxuening injection + Digoxin		30 minutes		Neiguan (PC6) Shenmen (HT7) Jueyinshu (BL14) Danzhong (RN16) Xinshu (BL15) Geshu (BL17)	
Li Chen (2012)	7 ± 3 m	8 ± 2 m	Paroxysm Persistent				Needle pricking		Amiodarone		24 hours		Neiguan (PC6)	
Hongke Xu (2007)	21.5 ± 23.9 hours	20.6 ± 22.9 hours	Paroxysm				Needle pricking + Amiodarone		Amiodarone		60 minutes		Neiguan (PC6) Danzhong (RN16) Qihai (RN6) Zhongwan (RN12) Zusanli (ST36)	
Xuelian Zhang (2013)	10.3 ± 5.2 years	11.2 ± 4.8 years	Paroxysm				Needle pricking + Wenxin particle		Wenxin particle		30 minutes		Neiguan (PC6) Shenmen (HT7) Ximen (PC4)	
Jieqiong Zhang (2020)	–	–	Paroxysm				Needle pricking + Nifekalan		Nifekalan		30 minutes		Neiguan (PC6) Shenmen (HT7) Ximen (PC4)	
Jianbao Wang (2022)	–	–	Paroxysm				Auricular acupoint Acupuncture		Shensongyangxin capsule		30 minutes		Neifenmi (C18) Shenmen (TF4) Jiaogan (AH6a) Shen (C10)	
Yang Chu (2023)	–	–	Persistent				Needle pricking + Catheter ablation		Catheter ablation		30 minutes		Neiguan (PC6) Shenmen (HT7) Ximen (PC4)	
Hewen Li (2023)	6.5 m (1.0, 16.5)	12.0 m (1.8, 43.5)	Paroxysm Persistent				Needle pricking + Catheter ablation		Sham-Acupuncture + Catheter ablation+		30 minutes		Xinshu (BL15) Neiguan (PC6) Shenmen (HT7)	
Xiaofei Gao (2023)	–	–	Paroxysm Persistent				Auricular acupoint Acupuncture + Catheter ablation + Amiodarone		Catheter ablation + Amiodarone		30 minutes		Xin (C15) Jiaogan (AH6a) Shenmen (TF4) Zhen (AT3) Pizhixia (AT4) Ermigen (R3)	
Albert Lomuscio (2011)	40 (34–43)m	39 (33–47)m	Persistent				Needle pricking + Catheter ablation + Amiodarone		Sham-Acupuncture + Catheter ablation + Amiodarone		15–20 minutes		Neiguan (PC6) Shenmen (HT7) Xinshu (BL15)	
Jung Myung Lee (2021)	–	–	Persistent				Electroacupuncture + Electrical cardioversion		Sham-Acupuncture + Electrical cardioversion		20 minutes		Jianshi (PC5) Neiguan (PC6) Shenmen (HT7) Zusanli (ST36) Shenmen (TF4)	
Yuanshi Xia (2014)	–	–	Paroxysm				Needle pricking + Digilanid C		Digilanid C		60 minutes		Neiguan (PC6) Ximen (PC4) Xuehai (SP10) Sanyinjiao (SP6)	

### 3.3. Assessment of the risk of bias

**Figure 2.**
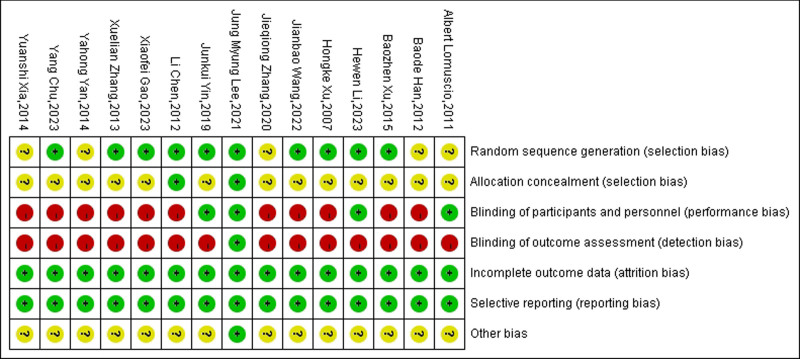
Risk of bias summary.

**Figure 3.**
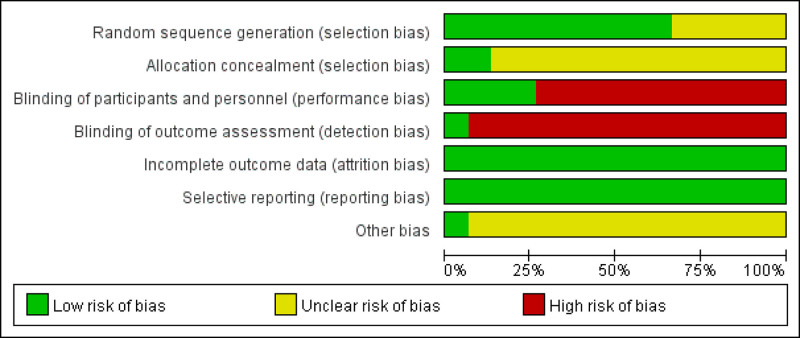
Risk of bias.

Among the included literature, ten articles reported a random method, all of which were random number table method, 4 reported a blind process, and 2 articles reported distribution concealment. Only 1 blinded 3 parties. The results of the risk of bias assessment, represented using Cochrane Review Manager software version 5.4 program, are shown in Figures 2 and 3: a summary of the risk of bias assessment for the included studies as Table 2.

**Table 2:** A summary of the risk of bias assessment for the included studies.

Study	Random sequence generation	Allocation concealment	Blinding of participants and personnel	Blinding of outcome assessment	Incomplete outcome data	Selective reporting
YuanShi Xia (2014)	?	?	+	-	+	+
Yang Chu (2014)	+	?	-	-	-	?
Xuelian Zhang (2020)	+	+	+	+	+	-
Xadrei (2019)	+	+	+	+	+	+
Li Chen (2012)	+	+	+	+	+	+
Jungkyul Yin (2019)	+	?	+	?	+	+
Jung Myung Lee (2021)	+	+	+	+	+	-
Jieqing Zhang (2020)	+	+	+	+	+	+
Hongke Xu (2023)	+	+	+	+	+	+
Baofen Hu (2012)	+	?	+	+	+	+
Baozhen Xu (2015)	+	+	+	+	?	+
Hewewen Li (2023)	+	+	+	+	+	+
Albert L. Lomuscio (2011)	+	?	+	-	+	?

+ indicates low risk of bias. - indicates high risk of bias. ? indicates unclear risk of bias.

### 3.4. Results of meta-analysis

#### 3.4.1. Sinus rhythm conversion rate

**Figure 4.**
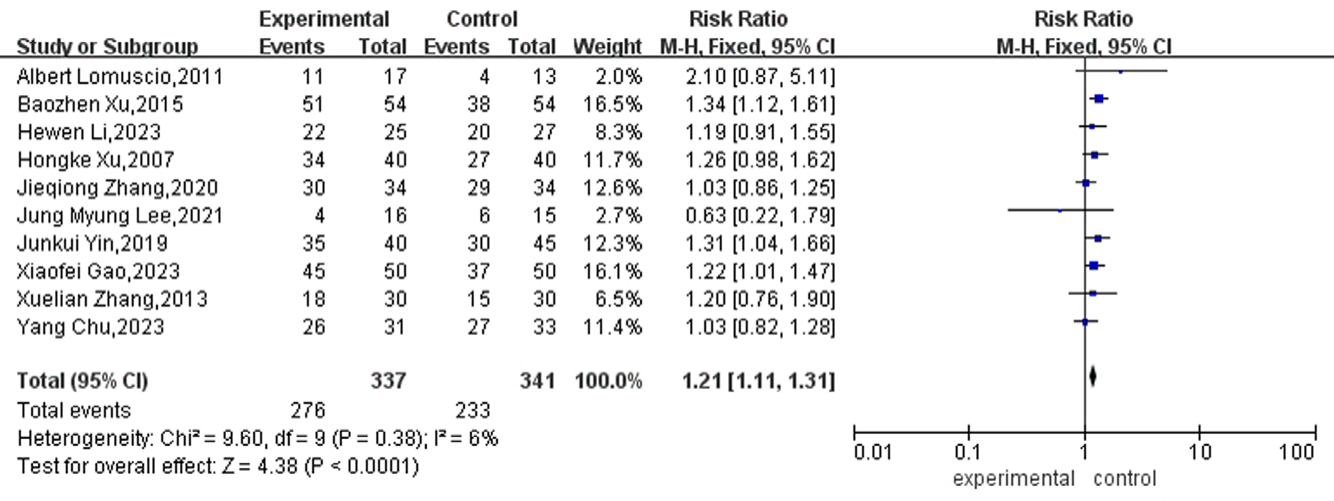
Forest map for sinus rhythm conversion ratio between the 2 groups.

Ten studies reported the therapeutic effect on the conversion of AF to sinus rhythm. Due to the absence of significant heterogeneity among these studies *(P* = .38, I² = 6%), a fixed-effects model was employed for the analysis. The results showed that acupuncture therapy could increase the sinus cardioversion rate of patients with AF compared with drug or CA therapy alone, and the difference was statistically significant (RR = 1.21, 25% CI [1.11, 1.31], *P* < .001). See Figure 4 for details.

**Figure 5.**
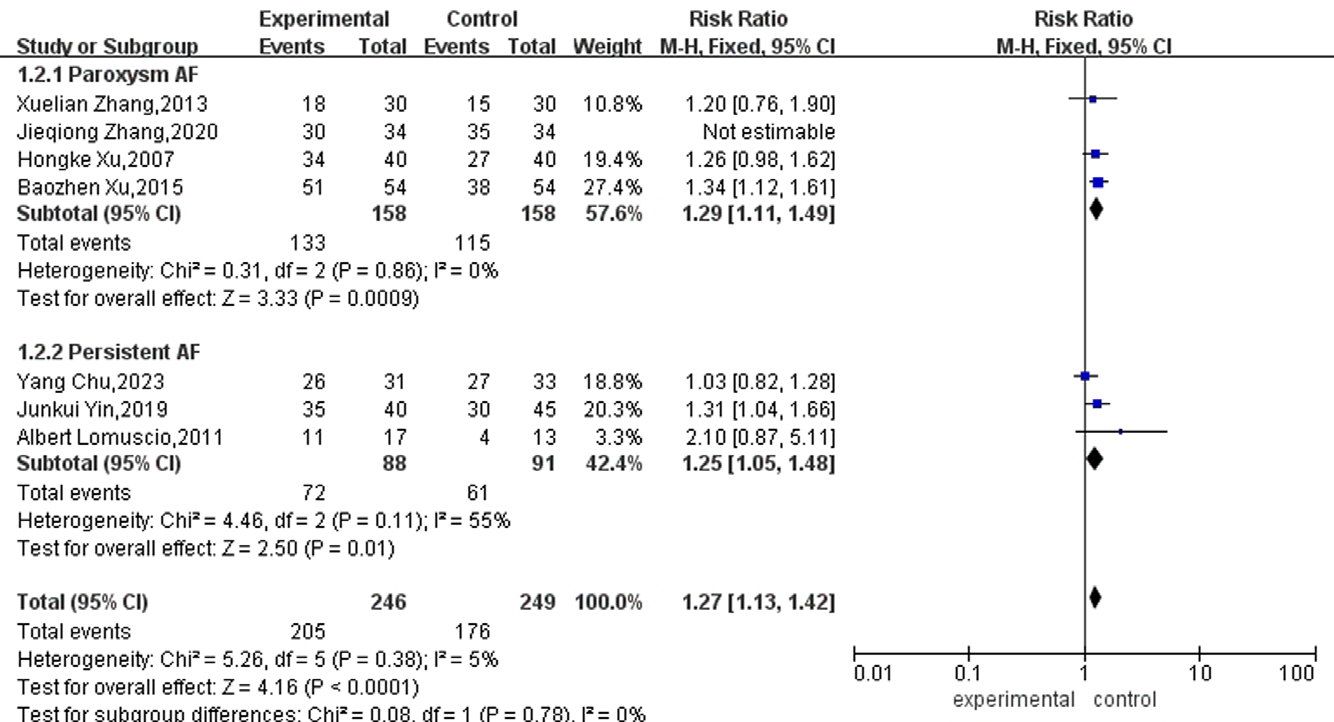
Forest map of subgroup analysis for the effect of acupuncture on different types of AF. AF = atrial fibrillation.

A subgroup analysis of the effect of acupuncture on paroxysmal or persistent AF was carried out. Due to the absence of significant heterogeneity among these studies (*P* = .38, I² = 5%), a fixed-effects model was employed for the analysis. The meta-analysis showed low heterogeneity between the 2 groups (I^2^ = 0%, *P* = .78), indicating that the type of AF does not affect the meta-analysis results. The results of 4 articles in the paroxysmal AF group showed that the adequate amount reached 1.21% and was significant, which means that acupuncture as an auxiliary therapy significantly increased the sinus rhythm conversion rate of paroxysmal AF. In the persistent AF group, fixed effects were selected, and the effect reached 1.27%, meaning that acupuncture and moxibustion as auxiliary therapy significantly increase persistent AF sinus rhythm conversion rate, as shown in Figure 5.

**Figure 6.**
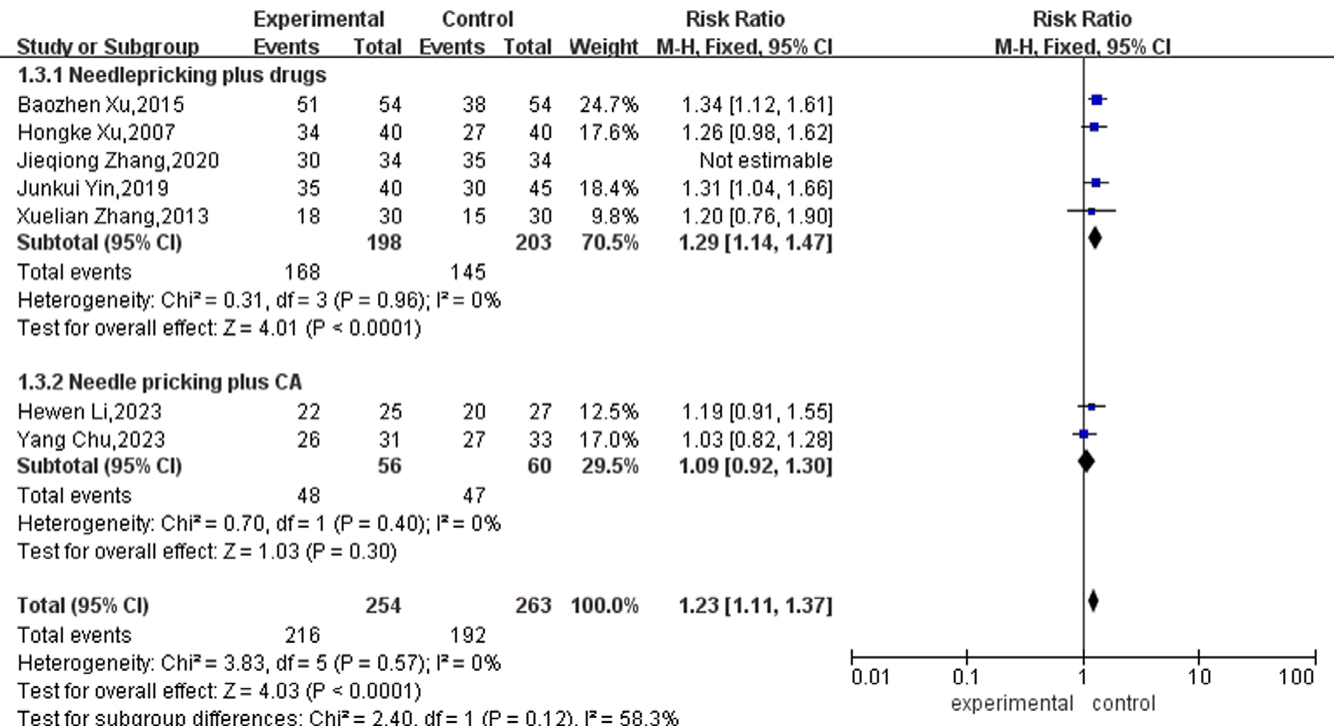
Forest map of subgroup analysis for the effect of acupuncture plus different treatment of AF. AF = atrial fibrillation.

A subgroup analysis of acupuncture plus different treatments for AF was carried out. Due to the absence of significant heterogeneity among these studies (*P* = .57, I² = 0%), a fixed-effects model was employed for the analysis. The meta-analysis showed moderate heterogeneity between the 2 groups (I^2^ = 58.3%, *P* = .12), indicating that acupuncture plus different treatment of AF could affect the meta-analysis results. The results of 5 articles using acupuncture plus drugs showed that the adequate amount reached 1.29% and was significant, which means that acupuncture as an auxiliary therapy significantly increased the sinus rhythm conversion rate after using drugs. In the acupuncture plus CA group, 2 studies were combined, and the effect reached 1.23%, which means that acupuncture as an auxiliary therapy dramatically increases the sinus rhythm conversion rate after CA, as shown in Figure 6.

#### 3.4.2. Clinical effective rate

**Figure 7.**
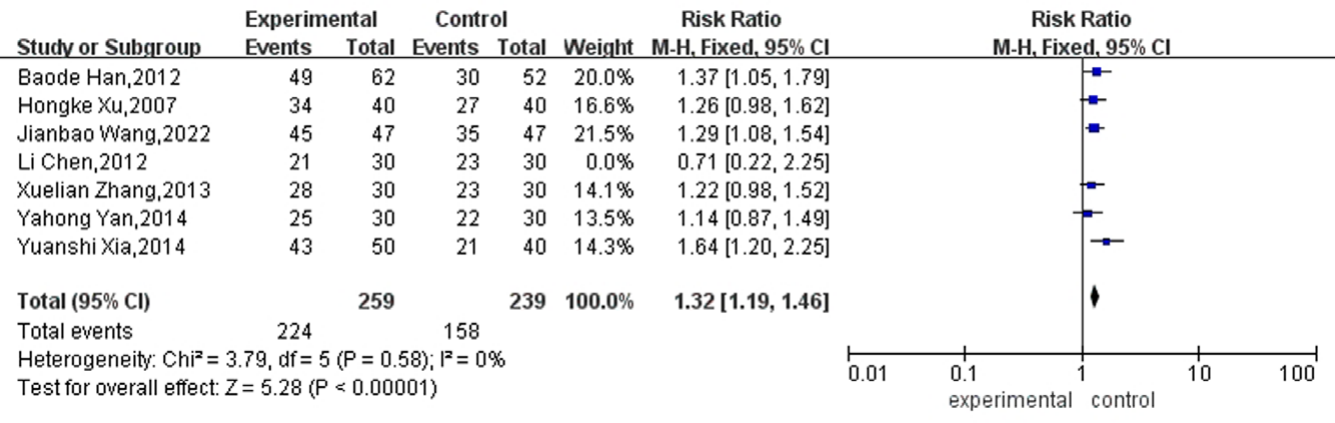
Forest map for clinical effective rate between the 2 groups.

Seven studies reported the clinically effective rate of acupuncture in treating AF. Heterogeneity test showed that I^2^ = 0%, *P* = .58. A fixed effect model was used for meta-analysis. The results showed that the clinically effective rate of the acupuncture plus drug treatment group was higher than that of the drug treatment group, and the difference was statistically significant (RR = 1.32, 95% CI [1.19, 1.46], *P* < .01], as shown in Figure 7.

**Figure 8.**
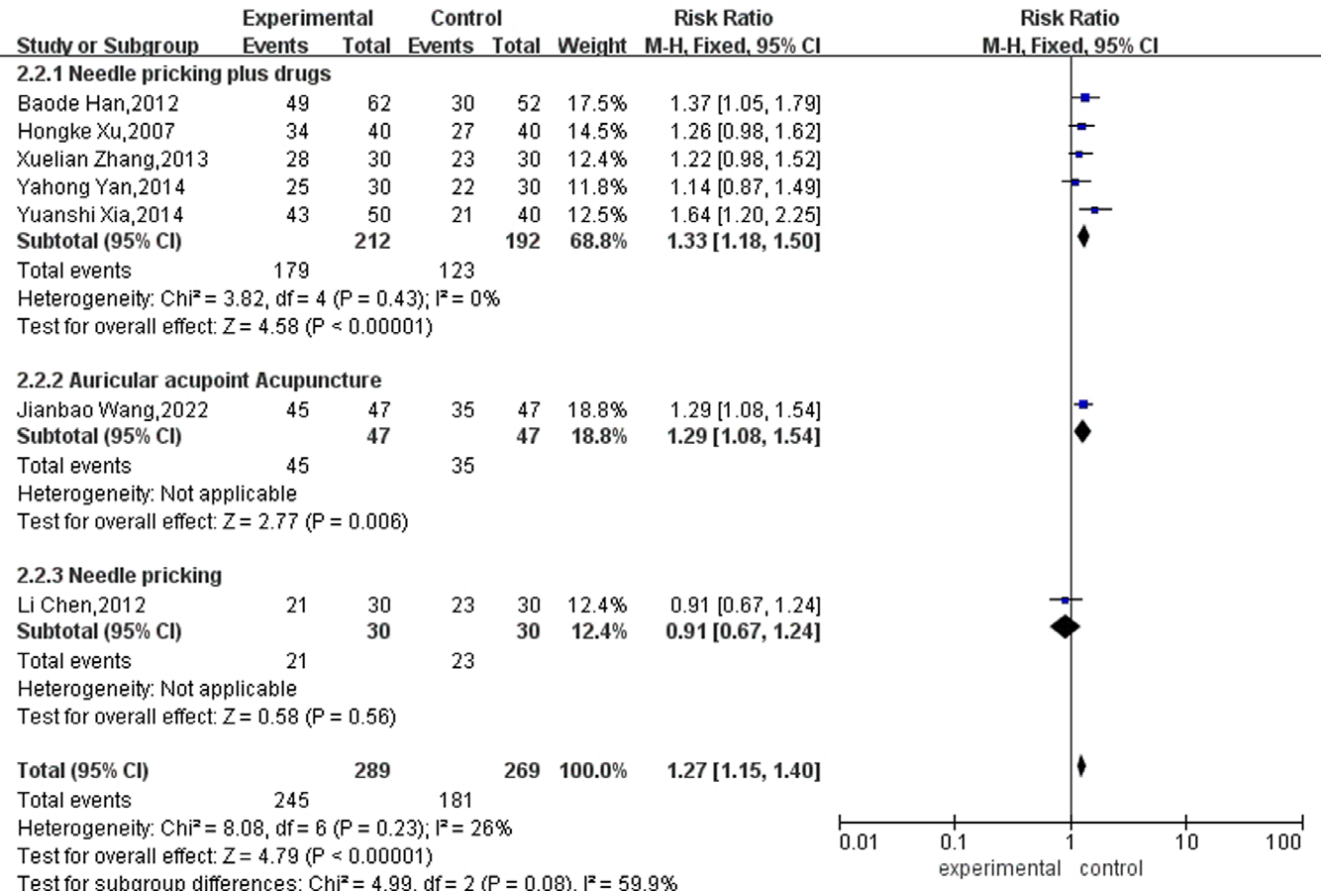
Forest map for clinical effective rate between the 2 groups.

A subgroup analysis of different types of acupuncture for clinical effectiveness rate was carried out. Due to the absence of significant heterogeneity among these studies (*P* = .23, I² = 26%), a fixed-effects model was employed for the analysis. The meta-analysis showed moderate heterogeneity between the 3 groups (I^2^ = 59.9%, *P* = .08), indicating that the different types of acupuncture could affect the meta-analysis results. The results of 5 articles using needle pricking showed that the adequate amount reached 1.33% and was significant, which means that needle pricking therapy significantly increased the sinus rhythm conversion rate. In the auricular acupoint acupuncture group, 1 study showed that the amount of effect reached 1.29%. Interestingly, in Li Chen analysis, the adequate amount of acupuncture alone was 0.91% of that of using medication alone, as shown in Figure 8.

#### 3.4.3. Ventricular rate

**Figure 9.**
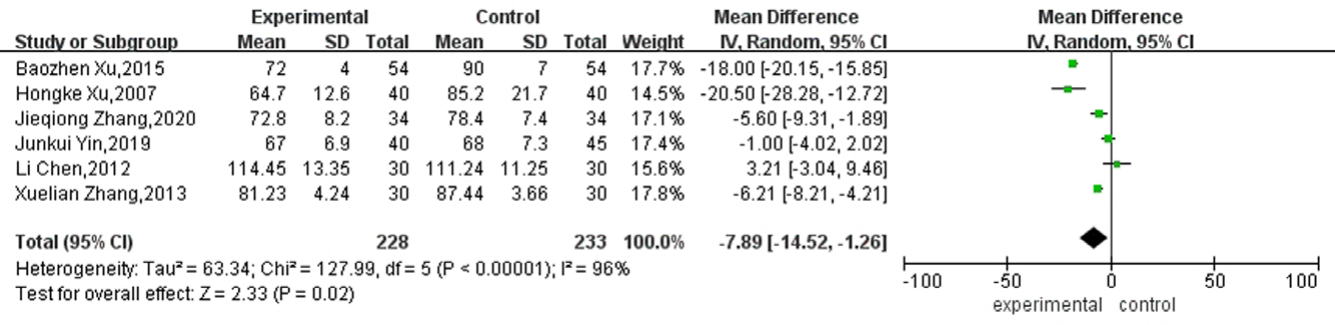
Forest map for ventricular rate between the 2 groups.

Six studies reported the effect on ventricular rate in patients with AF. Due to the significant heterogeneity among these studies (*P* < .38, I² = 96%), a random effect model was employed for the analysis. The results showed that acupuncture plus other conventional therapies treatment was more helpful in reducing the ventricular rate of patients with AF than traditional therapy alone, and the difference was statistically significant (MD = -7.89, 95% CI [-14.52, -1.26], *P* = .006). See Figure 9 for details.

#### 3.4.4. Conversion time

**Figure 10.**
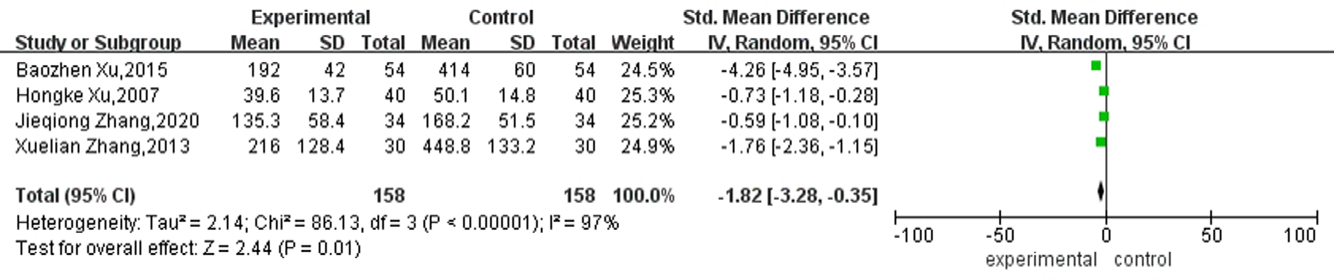
Forest map for conversion time between the 2 groups.

Four studies reported the effect on the conversion time to sinus rhythm in patients with AF. Heterogeneity test showed that I^2^ = 97% (*P* < .01). The random effect model was used to analyze the cardioversion time of the 2 groups. The results showed that the cardioversion time of patients with AF treated with acupuncture plus conventional therapies was shorter than that of patients treated with conventional therapies alone. The difference was statistically significant (SMD = -1.82, 95% CI [-3.28, -0.35], *P* = .01). See Figure 10 for details.

#### 3.4.5. Adverse reaction

A total of 7 articles in the study mentioned the problems of adverse reactions during the study. Zhang Xuelian reported that gastrointestinal reactions and bradycardia occurred in both groups during the study. Still, the number of cases in the test group was less than in the control group, and the above adverse reactions could be recovered by themselves. Zhang Jieqiong reported that acupuncture plus nifekalan and nifekalan alone had prolonged QT interval and bradycardia in the acupuncture group. Still, there was no significant difference in the incidence of adverse reactions between the 2 groups. Xu Hongke, Lomuscio, Lee, and Hewen Li all mentioned that compared with drug therapy alone, acupuncture did not cause adverse reactions such as bleeding, hematoma, and infection.

### 3.5. Publication bias

**Figure 11.**
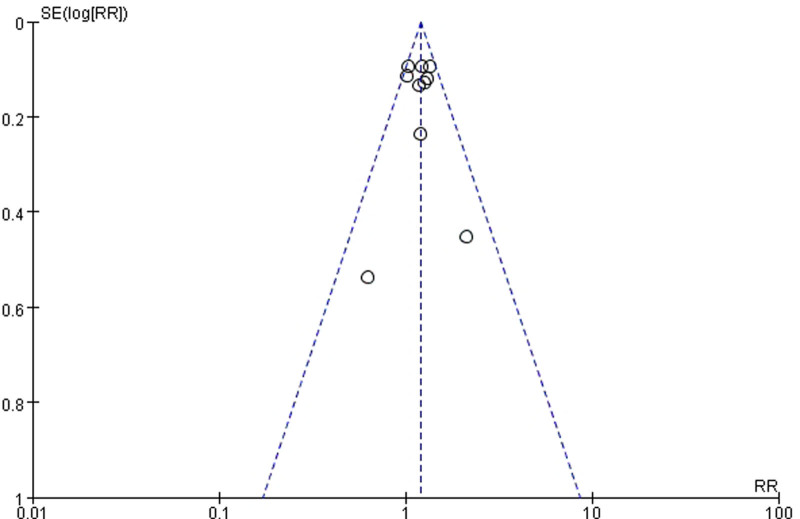
Funnel plot for sinus rhythm conversion rate.

**Figure 12.**
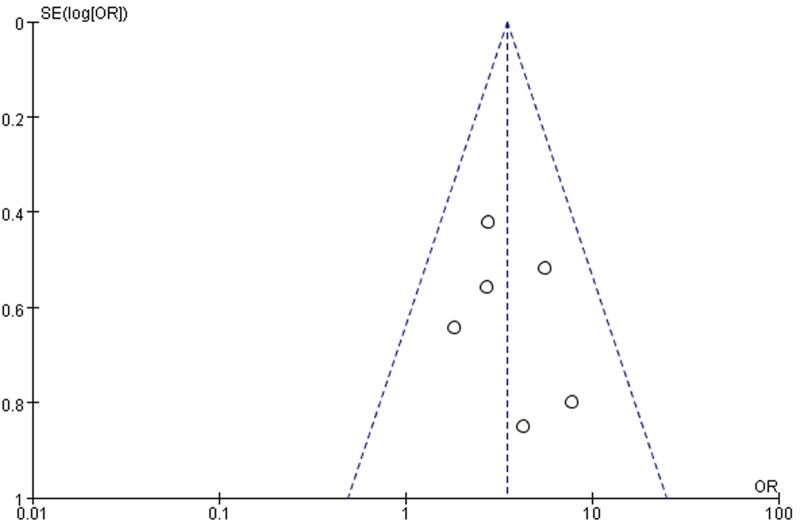
Funnel plot for clinical effective rate.

By drawing a funnel plot to examine whether there is publication bias in this study, the symmetry of the funnel plot means that there is no publication bias. The funnel plot of this study is shown in Figures 11 and 12. From the above figure, it is clear that there is no publication bias in the literature of this study, and the funnel plot is symmetric.

## 4. Discussion

### 4.1. Effect of acupuncture combined with other treatments on AF

This study analyzed data from 1960 patients in 15 studies. It was proved that combined with acupuncture based on drug, or CA antiarrhythmic therapy, it could improve the total clinical effective rate, increase the conversion rate of sinus rhythm, shorten the time to restore sinus rhythm, and reduce the ventricular rate of patients with AF. Among the 15 studies included, 10 were treated with acupuncture combined with antiarrhythmic drugs, 2 with CA, and 2 with simple acupuncture. The results showed that acupuncture was superior to non-acupuncture. It is suggested that acupuncture is effective in the treatment of AF as an adjuvant therapy or an alternative therapy. In addition, as an invasive treatment, the incidence of adverse reactions in acupuncture treatment was not higher than that in drug therapy alone, and acupuncture-related bleeding, hematoma, and infection were not reported. Therefore, on the whole, it can be considered that acupuncture is safe and effective for patients with AF based on treatment.

In the studies included in this study, acupuncture alone or acupuncture combined with antiarrhythmic drugs in controlling ventricular rate and rhythm in patients with paroxysmal AF. AF was better than that of antiarrhythmic medications alone. Xu Hongke et al^[26]^ found that acupuncture at Neiguan, Shenmen, and Qianzhong points had higher effects on the conversion rate of AF and ventricular rate in 90 minutes than those in the amiodarone group. The results of 2 prospective RCTs showed that acupuncture combined with lanatoside C in the treatment of paroxysmal rapid AF, the conversion rate of AF and the degree of ventricular reduction within 1 hour were better than those of lanatoside C alone.^[25,27]^ Yan Yahong et al^[24]^ showed that the frequency of AF in acupuncture combined with the routine treatment group was significantly lower than that in the routine treatment group. Xu Baozhen^[29]^ study found that acupuncture combined with Wenxin granule could increase the conversion rate of paroxysmal AF and reduce the ventricular rate and the levels of CRP and brain natriuretic peptide and BNP in blood.

Therefore, overall, when patients with AF are allergic to or resistant to antiarrhythmic drugs such as amiodarone, acupuncture can be used as an alternative therapy to help patients restore sinus rhythm. CA and electrocardioversion effectively convert sinus rhythm and improve quality of life in patients with AF, but they also have limitations. Complications such as thromboembolism, sedation-related complications, ventricular tachycardia (ventricular tachycardia), ventricular fibrillation (ventricular fibrillation), and chronic arrhythmias may occur in treating AF. The early recurrence rate after radiofrequency ablation is above 50%,^[36]^ and the late recurrence rate is 25% to 40%. The curative effect is unstable. Acupuncture can also effectively reduce the recurrence rate of AF after CA and electrocardioversion and improve adverse reactions such as sinus bradycardia.

### 4.2. Advantages of this study

Compared to previous meta-analyses, we strictly limited the inclusion criteria of literature and only included RCTs. We have made a more extensive exposition on the effect of acupuncture and moxibustion combined with drugs and radiofrequency ablation. The findings can potentially inform the clinical practice of AF by identifying more effective treatment strategies or interventions. Furthermore, a comprehensive understanding of the nuances and outcomes of various studies can contribute to the advancement of AF, enabling clinicians to tailor treatments based on specific findings and optimize efficacy for individual patients. Additionally, these findings may encourage the conduct of more long-term studies. The study may also inspire interdisciplinary research that combines insights from various fields to address complex health issues more comprehensively.

### 4.3. Limitations of this study

Despite strict inclusion and exclusion criteria, statistical heterogeneity was observed in many outcome indicators in this study. Key reasons for this include: (1) variability in primary diseases and treatments: patients had different primary conditions, such as coronary heart disease, dilated cardiomyopathy, and hypertension, leading to varied treatments like lipid-lowering drugs, myocardial nutrition drugs, and antihypertensive drugs. (2) Quality of included studies: most studies had low quality with unknown randomization methods. Due to the nature of acupuncture, blinding was rarely implemented. (3) Differences in treatment processes: variability existed in acupuncture location, duration, and selected acupoints. Neiguan was the most commonly used acupoint. Treatment duration ranged from single sessions to several weeks, and there were differences in techniques and needle retention times. (4) Non-uniform evaluation indices: the complexity of AF types (paroxysmal, persistent, and permanent) made it hard to use a unified evaluation index, and long-term follow-up was lacking. (5) Small sample sizes: most studies were small and mainly conducted in China, introducing potential language and regional biases. (6) Incomplete adverse event reporting: some studies only reported general adverse events without details on bleeding, hematoma, and local infections related to acupuncture. (7) Several antiarrhythmic agents that were assessed in RCTs and compared to acupuncture are not available in markets outside China, therefore, the efficacy is difficult to interpret.

These factors contribute to a high risk of bias (selective, implementation, measurement, and follow-up) and may affect the reliability of this meta-analysis. Thus, higher-quality RCTs are needed for more robust quantitative analysis.

### 4.4. Possible mechanism of acupuncture in treating AF

Studies have confirmed that acupuncture can treat AF through the above 4 pathways. It has been found that acupuncture at Neiguan point in rats with AF can reduce the expression level of connexin 40 in atrial tissue and damage atrial fibers and mitochondria. It can effectively protect the ultrastructure of the heart, delay the process of atrial structure remodeling, and reduce the mortality of rats.^[37–39]^ Gong Zhigang et al^[39]^ found that electroacupuncture at Hegu acupoint in dogs with acute AF could effectively inhibit the shortening of atrial effective refractory period caused by rapid atrial beat and the increase of atrial effective refractory period dispersion induced by AF. Then, it significantly alleviates the acute electrical remodeling of AF. Acupuncture at Lingtai and Shendao points out that patients with cardiac autonomic neuropathy can improve autonomic nervous activity and balance, change autonomic jumpy tension, and then improve cardiac function. In addition, acupuncture at Neiguan point in rats with AF could increase the expression level of calcium adenosine triphosphatase protein in atrial muscle and inhibit calcium overload. At the same time, it can reduce the irregular response of multiple waves caused by AF and increase the conduction velocity of electrical impulses between myocardial cells to reduce the duration of AF and the mortality of rats.^[40]^

### 4.5. Clinical relevance and implications for the future

The treatment of AF often varies based on the patient’s specific condition and individual differences. Similarly, acupuncture treatments in clinical practice should consider the individualized needs of patients, using the principles of traditional Chinese medicine to select the most appropriate acupoints and treatment methods. Acupuncture can serve as a non-pharmacological adjunct to conventional drug therapy, particularly for patients who are sensitive to medication side effects or who do not respond well to drug treatments. Furthermore, acupuncture may help reduce AF-related complications such as stroke and heart failure, thereby improving patients’ quality of life and survival rates.

Future research should aim to design higher-quality RCTs that ensure proper randomization and blinding to reduce bias and increase the reliability of the study results. Additionally, studies should establish standardized metrics for evaluating treatment efficacy, ensuring consistent methods for assessing different types of AF, which would facilitate comparison and comprehensive analysis of results. To better evaluate the long-term effects and safety of acupuncture, future studies should include long-term follow-up data, observing the impact of acupuncture on AF recurrence rates, severe complication rates, and patient survival. Expanding sample sizes through multicenter and cross-regional collaborative studies can reduce regional bias and enhance study results’ generalizability and external validity. Moreover, future research should meticulously record and report potential adverse events associated with acupuncture, such as bleeding, hematoma, and local infections, to provide a more comprehensive assessment of its safety. By implementing these improvements, we can better elucidate the potential role of acupuncture in the treatment of AF, provide more robust evidence to support its clinical application, and offer better healthcare services to patients.

## 5. Conclusion

To sum up, the systematic evaluation of the literature and the meta analysis of the curative effect data in this study may confirm that acupuncture may be used as an adjuvant therapy to effectively improve the total clinical effective rate and sinus rhythm conversion rate of patients with AF, shorten the time of conversion to sinus rhythm and reduce the ventricular rate. However, due to the limitations of research methodology and the quality of literature reports, the exact conclusion still needs to be further designed to be rigorous, large-sample, multicenter double-blind RCT evidence.

## Acknowledgments

We extend our sincere thanks to all the patients who participated in this study. Your willingness to contribute has been invaluable.

## Author contributions

**Conceptualization:** Xuemeng Pang.

**Data curation:** Yuqing Liu, Yajuan Wang.

**Formal analysis:** Yuqing Liu.

**Investigation:** Yuqing Liu, Xuemeng Pang.

**Methodology:** Yajuan Wang, Xuemeng Pang.

**Software:** Yuqing Liu.

**Supervision:** Xu Liu, Hongju Jiang.

**Visualization:** Yuqing Liu, Yajuan Wang.

**Writing – original draft:** Yuqing Liu, Yajuan Wang, Xu Liu.

**Writing – review & editing:** Yuqing Liu, Xuemeng Pang, Hongju Jiang.
